# Comparison of interdental papilla around single implants in the anterior maxilla between two implant systems: A cohort study

**DOI:** 10.15171/joddd.2018.006

**Published:** 2018-03-14

**Authors:** Masoumeh Khoshhal, Fariborz Vafaei, Mahsa Najafi, Masoumeh Nikkhah

**Affiliations:** ^1^Dental Implants Research Center, Hamadan University of Medical Sciences, Hamadan, Iran; ^2^Assistant Professor, Department of Prosthodontics, Faculty of Dentistry, Hamadan University of Medical Sciences, Hamadan, Iran; ^3^Dentist, Hamadan, Iran; ^4^Assistant Professor, Department of Periodontics, Faculty of Dentistry, Guilan University of Medical Sciences, Rasht, Iran

**Keywords:** Interdental papilla, maxilla, single-tooth implants

## Abstract

***Background.*** In successful replacement of a tooth with a dental implant, soft tissue esthetic is as important as stability and function of the implant. Quality and quantity of the peri-implant mucosa can influence esthetic outcomes. This study assessed implant esthetic success of two different implant systems. In this regard the interdental papilla was evaluated and the relation-ship between implant type and crestal bone loss adjacent to implant was assessed.

***Methods.*** Eighteen patients (11 males, 7 females) with a total of 18 implants participated in this historical cohort study. Patients were divided into two groups based on the type of implants: Implantium group and SPI group; 36 interproximal papillae were evaluated photographically, using Jemt’s papillary presence index (PPI). Radiographic analysis was carried out to find out the relation between bone loss and type of implant. Analysis of data was performed with SPSS 18, using Fisher's exact test, independent t-test, Spearman's correlation coefficient and ANOVA.

***Results.*** Comparison of photographs did not show a statistically significant difference in PPI between the two groups (P=0.94). Radiographic evaluation of crestal bone loss adjacent to implant shoulder did not reveal significant differences between the two groups (P=0.30).

***Conclusion.*** Implant therapy in the anterior maxilla, using Implantium or SPI system, did not result in significant differences in esthetics. In this study, there was an inverse relationship between the distance of contact point to bone crest and papilla index (P=0.002 in the SPI group) (P=0.02 in the Implantium group).

## Introduction

A maxillary anterior missing tooth is usually replaced for functional and aesthetic reasons.^1^

Implants are a quick way to replace teeth and achieve the desired results. Therefore, reconstructing the function and esthetic appearance by using a single-tooth implant compared to tooth-supported restorations has become a common treatment.

Although dental implants have long-term success in the reconstruction of edentulous patients, achieving desired beauty of soft tissue around anterior single implants and continued successful results is an equally important and concerned issue.

Gingival facial appearance and interproximal papilla are the most important factors that make a beautiful soft tissue.^2^

The interproximal space depends on several factors that can compromise the interdental papilla. Improper contours of restorations or prosthetic crowns, abnormal tooth shape, traumatic flossing and interproximal hygiene procedures and, importantly, periodontal diseases can cause recession in the interdental papilla.^3^

It is important to note that the marginal bone level can be maintained in the face of functional loading by a properly designed implant.^4^

The marginal bone level of the peri-implant and soft tissue are strongly related to each other, which determine the aesthetic outcome.^1^

Studies have shown that the height of papilla depends on the distance from the contact point to the crest of the alveolar bone of the adjacent teeth. On the other hand, anatomy of the adjacent teeth and the distance between the implants and natural teeth can affect the papilla formation.^5,6^

Although a number of studies have suggested that peri-implant bone level is a decisive factor for the presence of papilla between the tooth and implant, some studies have found that connective tissue attachment and clinical attachment level are also important.^7,8^

During a 6-month period, after implant surgical procedure, the level of soft tissue was enhanced considerably at tooth-facing sites (a mean of 1.1 mm) at proximal space, but no significant increase was found at implant-facing sites.^9^ Studies have shown differences between the level of the papilla in the mesial and distal aspects around single implant-supported restorations. The distal papilla has a lower score in Jemt classification compared to the mesial papilla adjacent to tooth surfaces.^5,10^ In 1997 Jemt^10^ reported that during a 1‒3-year period after single-implant restorations, the adjacent papilla regenerates partially without any clinical intervention. The reason for this spontaneous recovery of the papillae is unclear, but it

might be suggested that the inflammation caused by

accumulation of plaque in the proximal space leads to swelling of the soft tissue.

Won Lee et al^11^ showed that the height of the interproximal papilla between the single implant and the adjacent tooth is approximately equal to the interproximal papilla on the contra-lateral side. The alveolar bone loss on both sides of a single implant site would be similar.

A distance of 3 mm between two implants has been recommended to ensure the presence of papilla. On the other hand, some studies have shown that the interproximal papilla is not influenced by the distance between the implant and tooth.^12,13^

Several studies have been performed on the effect of the distance from the contact point of the implant restoration and the adjacent tooth to the crestal bone and the effect of implant design and diameter, implant position and soft tissue biotype on papilla around the implant.^14-17^

Many researchers have assessed the effect of different variables on peri-implant bone but have not considered its effect on soft tissues. However, evaluation of peri-implant tissues with different implant designs is mostly limited to assessing peri-implant bone level.^18,19^ The aim of this study was to compare and assess soft tissue around two commonly used implant systems (SPI and Impantium) with different designs with or without microthreads.

## Methods

In the present cohort study, 18 patients were selected among patients who had received single implants in the anterior area of the maxilla 1.5 years previously in a private clinic in Hamadan. The subjects were divided into two groups (n=9). The first group was treated by the Implantium system (Dentium, Korea) and the latter was treated by the SPI implant system (Thommen, Switzerland).

Features of these two implant systems were as follows: Body shape of these two types of implants was cylinderical. The abutment connection of these implants was internal and abutment connection type was internal hex. The implant body surface of the SPI system was acid etched, sandblasted and conditioned by hydroxylation. The implant body surface of Implantium was SLA (Sandblast Large grit Acid etch). The implant body thread of SPI was V-shaped and that of Implantium was buttress. The basic difference between these two implants systems was the presence of microthread in the neck of Implantium.

The inclusion criteria of the patients consisted of no systemic disease, no pregnancy, no smoking and drinking, no periodontal disease, good oral hygiene, no bleeding on probing (BOP) (BI=0), no pocket depths >3 mm, no bone loss (the distance between CEJ of the adjacent tooth and crestal bone was not >2 mm), implant in a safe distance from the adjacent tooth (at least 1.5 mm), thick gingival biotype, no history of radiotherapy and chemotherapy, and no use of immunosuppressive drugs. Exclusion criteria included uncontrolled active systemic disease, the need for bone grafts (GBR), thin gingival biotype, and prolonged steroid therapy.

In all the patients all the surgical procedures were performed by one periodontist and implant crowns were prepared by one prosthodontist. All the surgical and prosthetic procedures were the same. All PFM restorations were prepared by one technician and laboratory. All the implant restorations were cemented. All the patients had primary standard parallel periapical radiography. At the time of study, 1.5 years after implant insertion, standard parallel periapical radiography was obtained. To assess the presence of interproximal papilla, standard photographs was taken using a SLR camera (Canon 550D) with a 100-mm macro lens and ring flash.

In the initial radiograph, the upper edge of the implant shoulder and in the radiograph 1.5 years after the surgical procedure the distance between the implant and abutment was a considered reference line.

In the initial radiograph the distance from the contact point of the implant and bone to the reference line and also the distance from the CEJ of the adjacent tooth to the contact point of the crestal bone and tooth were measured. In the radiograph 1.5 years after implant insertion, the distance from the contact point of the implant restoration and the adjacent tooth to the crestal bone was measured. In addition, the distance from the contact point of the crestal bone and implant to the reference line and also the distance from the CEJ of the adjacent tooth to the contact point of the crestal bone and tooth were measured.

Presence of papilla between the implant and the adjacent tooth was measured and recorded based on Jemt index 1.5 years after implant insertion surgery. The distance from the height of the crestal bone and the implant shoulder was measured with a digital Vernier on initial the radiograph and the radiograph 1.5 years after surgery and recorded in mm. The distance between the height of the contact point of implant restoration and the adjacent tooth to crestal bone was measured using a digital Vernier on the radiograph 1.5 years after implant insertion surgery and recorded in mm. The distance between the CEJ of the adjacent tooth of implant to crestal bone was measured using a digital Vernier on the initial radiograph and the radiograph 1.5 years after surgery and recorded in mm.

All the measurements were performed by one person twice within two weeks and an average of the two measurements was used as the final measure.

The presence of papilla around implants was evaluated by photography and Jemt index.

Papilla index (PI) grading was as follows:

• Score 0: no papilla in the interproximal space

• Score 1: presence of less than 50% of the papilla height

• Score 2: presence of at least 50% of the papilla height but not all the interproximal space

• Score 3: the papilla completely fills the interproximal space and is coordinated by the adjacent papillae, with a favorable gingival contour

• Score 4: the hyperplastic papillae that covers too much of the single implant restoration and/or the adjacent tooth, with unfavorable gingival contour^20^

For this purpose, the line that connects the most apical point of the implant restoration to the most apical point of the crown of the adjacent tooth (zenith) was considered as a reference. The vertical distance from the contact point to this line was divided into two parts. Then the position of the papilla‏ tip was evaluated by Jemt index.

SPSS 18 was used for data analysis. To compare the presence of the interdental papilla in the two groups, Fisher's exact test was used. Independent t-test was used to compare the distance between the crestal bone and the CEJ of the adjacent teeth, implant shoulder, the contact point of the implant restoration and the adjacent tooth on the initial and secondary radiographs. Spearman's correlation coefficient and ANOVA were used to examine the relationship between different variables and the presence of papilla. Level of significance was set at P<0.05.

## Results

The mean age of the subjects was 40 years old, ranging from 26 to 55. Seven females and 11 males took part in the study. Totally 36 papillae and 18 interdental areas and papillae adjacent to implants were assessed in each group. A total of 12, 14 and 10 papillae were evaluated around canines, lateral incisors and central incisors, respectively.

The results in the two groups were matched in terms of the length and diameter of the implants.

Kolmogorov-Smirnov test showed that the distribution of all the data in Table 1 was normal. The mean changes in the distance between the crestal bone and implant shoulder in the mesial and distal aspects in the Implantium group were 0.68 mm and 1.31 mm, respectively with 1.09 and 0.7 mm, respectively, in the SPI group. The distance from the implant shoulder to the crestal bone increased significantly, only in the distal aspect of Implantium (P=0.008) (Table 1). The mean bone loss adjacent to the implant shoulder in the Implantium group was 1.32 mm, with 0.7 mm in the SPI group. Independent t-test did not reveal significant differences between the two groups (Table 2). Based on ANOVA there was no significant correlation between bone loss and papilla index in the two groups (Table 2). Kolmogorov-Smirnov test showed normal distribution of all the data in Table 3. As shown in Table 3 in the interval between implant insertion surgery and 18 months later, changes in bone level of the teeth adjacent to implant was not significant (Table 3). As shown in Table 4, there was no significant correlation between bone loss around the tooth adjacent to the implant in the mesial and distal aspects and papilla index in the two groups (Table 4). Based on Table 5, in both groups there was significant correlation between the distance from the contact point of the implant restoration and the adjacent tooth to crestal bone and papilla index. Correlation coefficients in the Implantium and SPI groups were -0.28 was -0.15, respectively, indicating an inverse relationship between the two variables. This means that by increasing the distance between the contact point and crest, the papilla index decreased (Table 5).

**Table 1:** Mean distances from the implant shoulder to the crestal bone in mm in the two groups

**Implant type**	**Zone**	**Initial radiography**	**After 18 months**	**The mean changes**	**Paired samples t-test**
**Implantium**	**Mesial**	1.21	1.9	0.68	P= 0.09
	**Distal**	0.79	2.11	1.31	P= 0.008
**SPI**	**Mesial**	1.95	3.05	1.09	P= 0.09
	**Distal**	1.76	2.47	0.7	P= 0.37

Significant level P> 0.05

**Table 2:** Bone loss adjacent to the implant shoulder and the relationship between bone loss adjacent to the implant shoulder and papilla index in the Implantium and SPI groups

	**Implantium**	**SPI**	**Level of significance**
**Mean ± SD**	1.32±0.97	0.70±1.41	P = 0.30
**F index in ANOVA**	4	3.2	P (Implantium) = 0.06
			P (SPI) = 0.08

Significant level P> 0.05

**Table 3:** The mean distance between the CEJ of the adjacent tooth and the crestal bone in mm in the two groups

**Implant type**	**Zone**	**Initial radiography**	**Secondary radiography**	**The mean changes**	**Level of significance**
**Implantium**	**Mesial**	2.22	2.56	0.33	P= 0.31
	**Distal**	2.21	2.31	0.09	P= 0.06
**SPI**	**Mesial**	2.21	2.67	0.46	P= 0.31
	**Distal**	2.67	2.80	0.12	P=0.68

Significant level P> 0.05

**Table 4:** The relationship between crestal bone loss of the adjacent teeth and papilla index in the mesial and distal aspects in the two groups

**Implant type**	**Zone**	**Level of significance based on variance analysis**
**Implantium**	**Mesial**	P = 0.41
	**Distal**	P = 0.77
**SPI**	**Mesial**	P = 0.35
	**Distal**	P = 0.29

Significant level P> 0.05

**Table 5:** The relationship between the distance from the contact point of the implant restoration and the adjacent tooth to crestal bone and papilla index

**Implant type**	**Spearman's correlation coefficient**	**Level of significance based on variance analysis**
**Implantium**	-0.28	P = 0.02
**SPI**	-0.15	P = 0.002

Significant level P> 0.05

## Discussion

Replacing lost teeth with implants is an ideal and successful treatment option. However, currently the main concern is not osseointegration of dental implants. Successful implant treatment means achieving the best esthetic results, in addition to stability and function of the implant.^21^

From an esthetic point of view presence and maintenance of interdental papilla is one of the main factors. In fact, esthetic results of implant treatment are not exclusively associated with the form of the crown, but they are mainly influenced by the topography of the surrounding soft tissue.

The design of this study was based on the recommendations of a study of Sorni-Broker et al, who suggested further studies to examine the effect of the micro- and macro-structure of the implant on the position of soft tissues, especially papilla.^22^

In this study, we compared interdental papillae in two different commonly used implant systems (Implantium and SPI). Implantium system has microthreads in the coronal area of the fixture and the SPI system does not have it.

The strength of this study in comparison with other studies was attention to interdental papilla as a key factor in the esthetic outcomes and success of implants.

It should be noted that most of the studies did not consider our subject and a small number of studies, such as studies by Jemt et al^31^ (2004) and Pier (2011), in which interdental papillae around different designs of implants were compared, had designs and variables different from those of the present study. Therefore, those studies cannot be directly compared with our study.

The results of this study did not reveal any significant differences in the amount of crestal bone loss around implants in the two groups with and without microthreads.

This is different from the results of studies by Lee et al,^23^ Nickening et al^24^ and Bratu et al.^25^ These studies suggested that microthreads in the coronal area of the fixture were effective in maintaining bone, and the amount of bone loss around these implants was significantly lower.

The differences between the results of this study and the study by Lee et al might be attributed to the study periods. Lee et al,^23^ during the 3-year study period, observed a significant difference between the groups with and without microthreads. However, in our study we evaluated the effect of different implants on papilla after 1.5 years of implant insertion surgery.

Nickening et al^24^ and Bratu et al^25^ used implants that were different from the types used in this study. They compared implants with and without microthreads (machined-neck implants). However, in the present study, implants with rough neck surface and with microthreads in one group, and implants with polished neck surface were used in the other group.

A study by Amid et al^26^ suggested that the implant neck with a microthread design relieved stresses in the bone crest. However, Schrotenboer et al^27^ suggested that microthreads increased crestal stress upon loading. These differences are explained by different implant systems and research methods of these studies.

Hansson^28^ suggested that, in the implant neck with a retentive feature, marginal bone loss will be prevented, decreasing peak interfacial shear stress. This study compared two different types of titanium implants by finite element analysis. Lee et al^29^ also observed that the implant with a rough surface and microthreads at the neck might have a chance of maintaining the level of the marginal bone. Differences in the results of the above studies and the present study might be explained by different types and surfaces of implants and different study periods.

The results of the present study are consistent with those of studies by Hartog et al^1^ and Shin et al.^19^ Hartog et al^1^ showed that after 18 months of follow-up, no considerable difference was found in the loss of marginal bone on radiographs between implants with smooth and rough necks. Shin et al^19^ reported no significant difference between rough-surfaced implants with and without microthreads in the amount of bone loss.

In this study there was no significant correlation between crestal bone loss adjacent to the implant shoulder and also between interproximal bone loss of the tooth adjacent to the implant and papilla index.

Studies by Nisapakultron et al,^30^ Hartog et al^1^ and Kan et al^16^ showed that the level of the interproximal bone crest of the adjacent tooth had a significant effect on the papilla level around the maxillary anterior single-tooth implants and was not very much related to bone level around the implant shoulder. This difference might be explained by the different designs of the studies.

Studies by Jemt et al^10^ and Henriksson et al^31^ showed that the distance of the contact point of the implant restoration and the adjacent tooth to the crestal bone had no significant effect on papilla presence. However, Choquet et al^14^ showed that when the distance between the contact point to the crest was ≤5 mm, the papilla was present almost in all the cases. When the distance was ≥6 mm, the papilla was present in half of the cases or less.

The results of this study were different from the observations reported by Jemt et al^10^ and Henriksson et al^31^ and the presence of a significant inverse relationship between the papilla index and the distance of the contact point of the implant restoration and the adjacent tooth.

This discrepancy might be attributed to different variables evaluated in studies by Jemt et al and Henriksson et al. They assessed the effect of different implant systems with different abutments on soft tissues.^31^

Overall, in the present study papilla formation in all the groups was acceptable, and none of them showed score 0. Frequencies of scores 2 and 3 in the two groups were the same. There was no significant difference in the amount of bone loss between the two groups. Therefore, both implant types exhibited almost good and comparable esthetic results in the anterior area of the maxilla.

## Conclusion

1. Presence of papilla and also the amount of bone loss adjacent to two implant systems did not exhibit significant differences.

2. There was a significant relationship between papilla presence and the distance of the contact point of the implant restoration and the adjacent tooth to the crestal bone.

3. There was no significant relationship between papilla presence and peri-implant bone loss and also bone loss around the adjacent tooth.

For future studies, it is suggested that a greater number of implants be included in each group for comparisons and also the effect of other types of implants on the interdental papilla be evaluated.

## Authors’ contributions

MKH presented the concept of the study. MKH and FV designed the study. MKH and FV were responsible for the intellectual content of the study. MN and MKH carried out the literature review. The clinical study was conducted by MKH and FV. MN was responsible for data acquisition and data analysis. The manuscript was prepared and edited by MN. The manuscript was reviewed by MKH and MN.

## Acknowledgments

This study was part of a thesis by Mahsa Najafi for a DDS degree. The thesis supervisor was Dr. Masoumeh Khoshhal and the counseling professor was Dr. Fariborz Vafaei.

## Funding

Hamadan University of Medical Sciences financially supported this study.

## Competing interests

The authors declare that they have no significant competing financial, professional or personal interests that might have influenced the performance or presentation of the work described in this manuscript.

## Ethics approval

This study was approved by the Medical Ethics and Research Committee of Hamadan University of Medical Sciences.
